# Effect of Ocean Acidification and pH Fluctuations on the Growth and Development of Coralline Algal Recruits, and an Associated Benthic Algal Assemblage

**DOI:** 10.1371/journal.pone.0140394

**Published:** 2015-10-15

**Authors:** Michael Y. Roleda, Christopher E. Cornwall, Yuanyuan Feng, Christina M. McGraw, Abigail M. Smith, Catriona L. Hurd

**Affiliations:** 1 Department of Botany, University of Otago, Dunedin, New Zealand; 2 Department of Chemistry, University of Otago, Dunedin, New Zealand; 3 Department of Marine Science, University of Otago, Dunedin, New Zealand; 4 Institute for Marine and Antarctic Studies, University of Tasmania, Hobart, Tasmania, Australia; The Evergreen State College, UNITED STATES

## Abstract

Coralline algae are susceptible to the changes in the seawater carbonate system associated with ocean acidification (OA). However, the coastal environments in which corallines grow are subject to large daily pH fluctuations which may affect their responses to OA. Here, we followed the growth and development of the juvenile coralline alga *Arthrocardia corymbosa*, which had recruited into experimental conditions during a prior experiment, using a novel OA laboratory culture system to simulate the pH fluctuations observed within a kelp forest. Microscopic life history stages are considered more susceptible to environmental stress than adult stages; we compared the responses of newly recruited *A*. *corymbosa* to static and fluctuating seawater pH with those of their field-collected parents. Recruits were cultivated for 16 weeks under static pH 8.05 and 7.65, representing ambient and 4× preindustrial *p*CO_2_ concentrations, respectively, and two fluctuating pH treatments of daily $\tilde{x}\;=\;8.05$ (daytime pH = 8.45, night-time pH = 7.65) and daily $\tilde{x}\;=\;7.65$ (daytime pH = 8.05, night-time pH = 7.25). Positive growth rates of new recruits were recorded in all treatments, and were highest under static pH 8.05 and lowest under fluctuating pH 7.65. This pattern was similar to the adults’ response, except that adults had zero growth under fluctuating pH 7.65. The % dry weight of MgCO_3_ in calcite of the juveniles was reduced from 10% at pH 8.05 to 8% at pH 7.65, but there was no effect of pH fluctuation. A wide range of fleshy macroalgae and at least 6 species of benthic diatoms recruited across all experimental treatments, from cryptic spores associated with the adult *A*. *corymbosa*. There was no effect of experimental treatment on the growth of the benthic diatoms. On the community level, pH-sensitive species may survive lower pH in the presence of diatoms and fleshy macroalgae, whose high metabolic activity may raise the pH of the local microhabitat.

## Introduction

Rising CO_2_ emissions are lowering the pH of the world’s ocean [1,2]. If anthropogenic emissions continue unabated, the present average surface seawater pH of 8.1 is projected to drop to 7.8 by 2100 [1]. The projected drop in pH (i.e. increase in proton [H^+^] concentration) results in changes to the seawater carbonate system, termed ‘ocean acidification’ (OA). OA could cause wide-ranging effects to marine ecosystems, by affecting individual organisms at all trophic levels, from bacteria to fish, and therefore ecosystem functioning [3–8]. Calcifying organisms are particularly susceptible, because perturbations in the seawater carbonate system, including changes in [H^+^] and [CO_2(aq)_], can reduce their ability to synthesise and/or maintain calcium carbonate skeletons [9–11].

Coralline algae are calcifying red seaweeds (Rhodophyta) that dominate benthic coastal waters from tropical to Polar regions, providing essential ecosystem services including structural frameworks and carbonate deposition [12]. They are considered the most susceptible of all calcifiers to OA [13]; most laboratory/mesocosm studies reveal reduced rates of growth and/or calcification [14,15] and field studies along natural pH gradients in volcanic vent sites show reduced abundances in sites with lower pH [16–18]. The majority of studies on coralline algae have been conducted in tropical and warm temperate regions (e.g. [16–19]), with fewer studies on cold temperate species (e.g. [14,15,20]). This is surprising, because cold temperate regions and the Polar seas are projected to be more vulnerable to OA because cold water absorbs more CO_2_ [21,22].

In cold-temperate systems of the northern and southern hemispheres, coralline algae grow in coastal waters within which strong diel, semi-diurnal and stochastic pH oscillations of varying amplitudes have been reported [14,23]. The pH variations are associated with biological activity, with photosynthesis causing pH to increase during the day and respiration causing pH to decrease at night [24]. Coralline algae are often associated with canopy-forming seaweeds, especially members of the orders Fucales and Laminariales (termed ‘kelps’). The kelps are responsible for changing pH in the surrounding water, for example, pH in kelp forests exhibits large diurnal fluctuations changing > 0.25 units [23]. Coralline algae themselves can also metabolically modify pH at their surface, within the diffusion boundary layer [20,25]. Therefore, coralline algae, and other associated benthic organisms growing within or near coastal kelp forests are naturally exposed to a daily cycle of both high (~8.86) and low pH (~7.7) [14,23].

The strong pH fluctuations of coastal kelp forest ecosystems changing > 0.25 units, are in sharp contrast to those observed in the open ocean, where fluctuations range from 0.024 to 0.096 [23]. However, the majority of laboratory experiments testing the effects of OA on benthic coastal organisms have been made in experimental systems that maintain a relatively constant pH or in systems where pH is allowed to vary naturally but is not controlled [26]. An exception is Cornwall et al. [14] who rigorously controlled pH and found that pH fluctuations negatively affected the growth rates of an articulate coralline alga, *Arthrocardia corymbosa*, although no other physiological diagnostics (e.g. photosynthetic efficiency, pigment content and tissue elemental composition) were affected by pH or pH fluctuations. Therefore, pH fluctuations can affect the outcome of experiments examining organismal responses to OA.

The majority of experiments testing the effects of OA on seaweed have examined adults, with relatively few studies on juveniles [27,28]. This is surprising because juveniles are considered the most susceptible life history stage to environmental stress [29–32]. Here we investigate the effects of static vs fluctuating pH on the growth and development of new recruits of the coralline alga *Arthrocardia corymbosa* (Lamarck) Decaisne (hereafter *Arthrocardia*).

**Fig 1:**
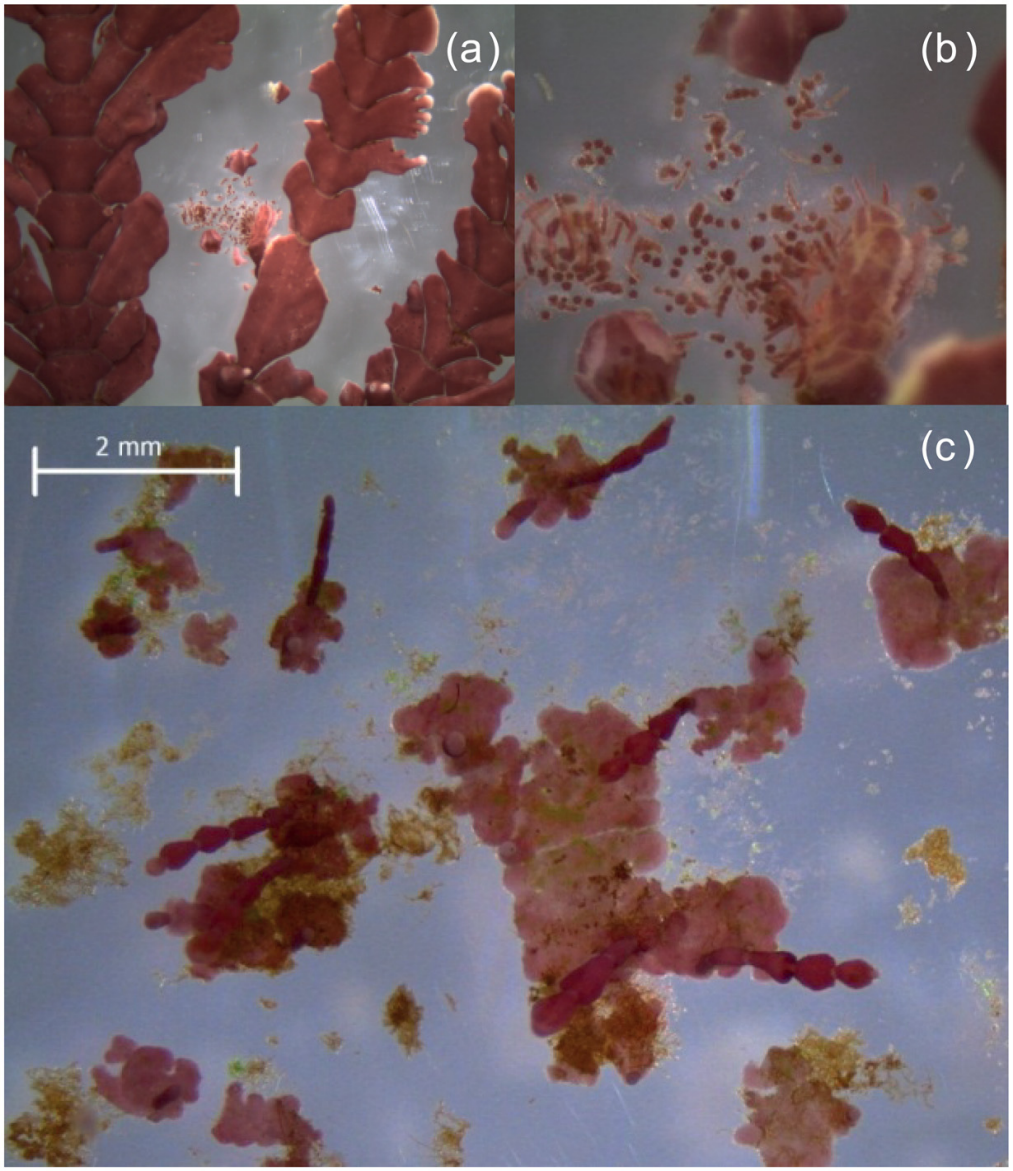
The articulate coralline *Arthrocardia corymbosa*. (a) Reproductive sporophyte, (b) Tetraspores, and (c) Germinating spores initially coalescing into an extensive crustose holdfast, which subsequently develop into upright articulated fronds.

This experiment is a continuation of Cornwall et al. [14], who grew field-collected mature adults of *Arthrocardia* (Fig 1a) under (1) static pH 8.05 representing the current average global surface water pH; (2) a diurnally fluctuating pH, with a daytime pH of 8.45 and night-time pH of 7.65 (daily $\tilde{x}\;=\;8.05$); (3) a static pH of 7.65, which represents the projected ‘worse-case scenario’ of a 0.4 unit reduction in pH over the next century; and (4) a pH that was fluctuated diurnally around the average surface seawater pH projected for 2100, with a daytime pH of 8.05 and night-time pH of 7.25 (daily $\tilde{x}\;=\;7.65$). During the Cornwall et al. [14] experiment, the adult *Arthrocardia* released spores onto the Perspex plates to which they were attached. Here we follow the growth and the development of these spores to crusts and upright thalli, under the same experimental conditions as Cornwall et al. [14], for a further 16 weeks. We hypothesized that (1) coralline recruit growth rates in the low pH treatments (both static and fluctuating pH 7.65) would be lower than rates under ambient pH treatments (both static and fluctuating pH 8.05), (2) growth rates of coralline crusts under fluctuating pH will be lower than those under static pH (*cf* [14] for adults), and (3) there would be no change in the % weight of Mg Calcite between experimental treatments, as observed for adults (*cf* [14]). Also, because the juveniles were recruited from adults that had already been in experimental culture for 6 weeks, we were able to make broad comparisons between the growth and mineralogical responses of the adults and juveniles to pH and pH fluctuation treatments. During this experiment, a number of benthic diatoms and fleshy seaweeds also recruited, from cryptic spores associated with the mature *Arthrocardia*, and these were identified and enumerated. For the diatoms, we developed an *a posteriori* hypothesis at week 7 that diatom biomass will be comparable under all experimental treatments because elevated CO_2_ would neither benefit nor negatively affect diatom growth rate [7].

## Materials and Methods

### Algal material

Field material of *Arthrocardia* was collected using permits provided by the New Zealand Ministry of Fisheries to the University of Otago. The field site is not a marine reserve, nor is *Arthrocardia* classified as endangered.

This experiment is a continuation of Cornwall et al. [14], in which ‘clumps’ of mature *Arthrocardia*, collected on 12^th^ of March 2011 from under a canopy the giant kelp *Macrocystis pyrifera* at 1.5 to 2 m depth in Karitane, Coastal Otago, New Zealand (45° 38' 20" S, 170° 40' 15" E), were cultured under the static and fluctuating pH treatments (described below) for 6 weeks; twenty-four clumps of *Arthrocardia*, each composed of 10 upright thalli standing on a small crustose holdfast, were secured onto each of 24 circular Perspex plates (70 mm diameter) using nylon fishing line. At the end of Cornwall et al. [14] visible post-settlement recruits were observed on the Perspex plate under and around each articulate algal canopy; the adult *Arthrocardia* were removed from the culture system on April 24^th^ 2011 [14], the Perspex plates were photographed then returned immediately to their respective daytime experimental pH conditions. In this study, the development of these coralline algal recruits was followed for 16 weeks, until August 13^th^ 2011.

### Experimental design: automated pH-controlled culture system

Seawater (salinity 34.3 *S*_*A*_) used during the experiment was collected from Portobello Marine Laboratory, University of Otago, located in Harrington Point at the entrance of Otago Harbour. The water is well flushed and receives typical levels of coastal nutrients [33] and is not influenced by heavy metal contamination [34]. Before use, seawater was filtered through Filter Pure^®^ polypropylene spun cartridge (5 μm pore size) and ultraviolet sterilized with an Aquastep^®^ 25 watts Ultraviolet Sterilizer. Initial concentrations of nitrate and phosphate were 2.302 ± 0.048 and 0.179 ± 0.008 μM, respectively.

The 24 plates upon which the corallines had recruited were grown in a flow-through Plexiglas^®^ acrylic culture tank (650 mL) following the assigned random pH treatments (n = 6) of [14]. The mean daily pH treatments were two static pH levels (pH_T_ 8.05 and 7.65) representing present day and the worst case scenario future ocean under OA, respectively, and two diurnally fluctuating pH treatments in which the mean surface ocean present day pH (8.05) and future pH (7.65) was increased by 0.4 units during the day and decreased by 0.4 units a night: accordingly, daytime pH was 8.45 and 8.05, and night time pH was 7.65 and 7.25, for the present and future ocean (2100) conditions, respectively. The 0.8 unit difference between night and day time pH is comparable to the amplitude of summer pH fluctuations observed in the field [14].

The pH treatments were achieved using a modified version of the automated pH-controlled culture system described by [35]. This system was housed in a walk-in growth chamber at 10.8°C under a 12:12 h light/dark cycle and a mean irradiance of 18 μmol photons m^-2^ s^-1^ which is optimal for the growth of these coralline algae [14,15]. Briefly, the ambient seawater pH_T_ 8.01 ± 0.02 was increased to pH_T_ 8.45 by using 0.5 M NaOH, and stored in a covered 150 L storage tank for further pH manipulation using the automated system. Seawater was drawn from the storage tank into the 1 L mixing tank and target pH_T_ levels were achieved by adding 0.5 M NaHCO_3_ and 0.5 M HCl. This method of pH adjustment results in changes to the carbonate chemistry and total alkalinity (A_T_) that are chemically identical to CO_2_ bubbling [36,37]. After mixing, pH_T_ was spectrophotometrically measured using indicator dye. When the pH_T_ level was within 0.03 units of the target pH, the seawater was transferred to the appropriate 1 L header tank. Both the mixing and header tanks were air-tight. If seawater pH adjustment exceeded the 0.03 pH unit tolerance level, seawater in the mixing tank was sent to waste and the process repeated until the desired pH and tolerance level were achieved. Once filled, the pH-adjusted seawater from the header tank automatically supplied fresh medium to its respective 650 mL culture tank. The inflow was located at the bottom while the outflow was at the top of the air-tight culture chambers. The automated system required approximately 4.4 hours to complete one cycle of delivering seawater to all of the 24 header tanks. The order of exchanging pH-adjusted seawater into each culture chamber was determined at random. To maintain seawater supply for the semi-continuous flow-through culture system, seawater in the storage tank was replenished 2× a day. To minimise the thickness of the diffusion boundary layer at the surface of the organisms, culture chambers were provided with water movement using magnetic bars under the Perspex plate, stirred at 550 rpm. This rpm provides an an instantaneous seawater velocity of 4.3 cm s^-1^. The seawater velocities were previously measured in the culture chamber using a Nortek Ventrino micro-Acoustic Doppler Velocimeter (micro-ADV; C.A. Pilditch, University of Waikato, New Zealand). The micro-ADV was placed in the culture chamber and then the velocity measured for 120 s, at 25 Hz, with the stirrer bar set at 550 rpm.

### Coralline algal growth

The size of the coralline algal recruits that grew initially as crusts was quantified on two occasions, on the 26^th^ June and 13^th^ August 2011. Recruits on the acrylic plates, submerged in seawater, were observed under a Leica EZ4 D stereo microscope. Colour images were captured using the built-in digital 3MP camera and processed using the LAS EZ software for PC (Leica Microsystems, Wetzlar, Germany). Coralline algal recruit surface area was then measured using image processing software (ImageJ 1.46; http://imagej.nih.gov/ij/). Photos taken from both time points were compared and the same ten individual haphazardly selected coralline algal recruits were identified. Relative growth rates, μ, of each recruit disc were calculated as:

$$\mu \;=\;\frac{ln(\frac{{D_{t}}_{2}}{{D_{t}}_{1}})}{t_{2}-t_{1}}$$

where *D*_*t*_ is disc size at different time points (*t*_1_ and *t*_2_), respectively. Average μ was calculated per culture tank (10 individuals) and treatments (n = 6, ± SE). In addition, the number of upright thalli that developed from the crusts between the 26^th^ of June and 13^th^ of August were counted.

### Skeletal mineralogy: x-ray diffractometry

At the end of the experiment, crusts and erect thalli of coralline macroalgae were removed from the plates by scraping with a scalpel. Samples were bleached to remove organic material, rinsed and dried. They were ground to a fine powder in an agate mortar with 0.1 g NaCl as an internal XRD standard, spread out and dried on a glass slide to randomize crystallite orientation. Each slide was run through a PAN Analytical X'Pert PRO X-ray diffractometer at a scan speed of 0.02571 °2 θ, over the range of 26 to 33 °2 θ. Peak heights (in counts) and positions (in °2 θ) were determined using X'Pert Data Collector and High Score data processing. The halite peak position was standardized to 31.72 °2 θ, and other peak positions corrected. The percent Mg in the calcite by dry weight was calculated from calcite peak position (in °2 θ) using the equation y = 30x−882 [38]. Each spectrum and the locations of ragged peaks were visually inspected and confirmed. Relative peak height counts (ht) of aragonite (A1 at 26.213 °2 θ and A2 at 27.216 °2 θ) and calcite (C1 at 29.4 to 29.8 °2 θ) were used to calculate Peak Height Ratio (PR) for each graph: PR = (ht A1 + ht A2)/(ht A1 + ht A2 + ht C1). Wt% calcite was calculated using the calibration of [39]: Wt % Calcite = 80.4 (PR)^2^–180.9 (PR) + 101.2. This method assumes that only calcite and aragonite are present.

### Observations on ‘fleshy’ macroalgal recruits

During this experiment, a number of non-calcifying seaweeds recruited onto the Perspex plates. The identity and number of these juvenile fleshy macroalgae on each Perspex plate was recorded on day 60 and again on the final day of the experiment (day 111).

### Benthic diatoms

Benthic diatoms were observed growing around the Plexiglas walls of each culture vessels and on the surface of the Perspex plate. Diatom films around the Plexiglas walls were gently removed with a soft-bristle paintbrush every 2 weeks to avoid dense cover, and in conducting this removal, we were able to quantify the biomass of diatoms that accumulated every 2 weeks. The result of the brushing was that the diatom cells were suspended within in each culture vessel. These were decanted and the seawater immediately replenished with newly pH-adjusted seawater stored in the header tank. Diatom biomass within a 2 ml aliquot removed from the 650 ml cell suspension was quantified every 2 weeks from 3^rd^ June 2011 to 13^th^ August 2011. The 2 mL aliquot of the cell suspension from each culture vessel was fixed in glutaraldehyde at 1% final concentration. Cells were counted and identified under a Zeiss microscope (Axiostar plus). For species identification, sets of 0.5 mL of aliquots were filtered onto 0.6 μm pore size polycarbonate filters (Whatman) under low pressure of vacuum. The filters were then air-dried on a plastic Petri-dish and examined using a scanning electron microscope (JEOL Ltd. Tokyo, Japan) after being coated with gold.

For the quantification of benthic diatom chlorophyll *a* (Chl *a*), 10 mL of the cell suspension were filtered under a low vacuum (30–40 mm Hg) onto Whatman GF/F glass fiber filters. Pigment was extracted in 90% acetone at 4°C in dark for 18 hours, and measured using a Turner 10-AU fluorometer [40]. Due to variable cell densities and different species’ composition in each culture tank, biomass normalized Chl *a* concentration was standardized with particulate organic carbon (POC) concentration and expressed as μg Chl *a* (μg C)^-1^.

For particulate organic carbon (POC) and nitrogen (PON) analysis, sample volumes of 20 ml were collected onto pre-combusted (450°C, 2 hours) Whatman GF/F glass fiber filters, dried at 55°C, and wrapped in tin capsules. Molar POC and PON were quantified after ignition in a Costech Elemental Combustion System (Costech Analytical Technologies Inc., Valencia, CA, USA), calibrated with EDTA (C:N = 4.29) and phenylalanine (C:N = 7.72) as reference materials.

For particulate organic phosphorus, sample volumes of 10 mL were collected onto pre-combusted (450°C, 2 hours) Whatman GF/F glass fiber filters. These were rinsed with 2 mL 0.17 mol L^-1^ Na_2_SO_4_ solution, placed in 20-mL pre-combusted (450°C, overnight) borosilicate scintillation vials with 2 mL 0.017 mol L^-1^ MgSO_4_ added. The liquid was evaporated to dryness at 95°C. For analysis, the vials were combusted at 450°C for 2 hours. After cooling, 5 mL of 0.2 mol L^-1^ HCl was added. The vials were then tightly capped and heated at 80°C for 30 minutes for digestion. Dissolved phosphate concentration from the digested particulate organic phosphate (POP) sample was measured colorimetrically using a spectrophotometer as described in [41].

For biogenic silica (BSi), sample volumes of 20 ml were collected onto 0.6 μm 47 mm polycarbonate filters, dried at 60°C, and then stored in a dessicator at room temperature until analysis. BSi was measured following the method of [42]. The BSi quota was standardized by Chl *a* concentration and expressed as μmol (μg Chl *a*)^-1^.

### Effect of community metabolism on seawater carbonate parameters

To determine if the algal assemblage modified seawater pH and alkalinity, these two parameters were quantified on the 55^th^ day of the experiment (June 18^th^ 2011). For the carbonate chemistry measurements, ambient seawater (pH 8.01), pH-adjusted seawater (initial pH 7.25, 7.65, 8.05, and 8.45), and after 4.4 h incubation during the day (18 μmol photons m^-2^ s^-1^ photosynthetically active radiation, PAR) from the initial pH (pH 7.65, 8.05, and 8.45) corresponding to each treatment were collected and fixed in mercuric chloride. Briefly, at the end of the 4-hourly seawater exchange cycle in our close system, 500 mL seawater from each culture tank was collected, just before the newly manipulated seawater stored in the header tank was allowed to replenish seawater in each 650 ml culture vessel.

### Seawater chemistry

Total alkalinity (A_T_) of samples was measured using the closed-cell titration method described by [43]. A_T_, pH_T_, salinity, and temperature were used to calculate carbonate chemistry parameters using the program SWCO2 [44].

### Data analysis

The effect of mean pH (ambient/low), diurnal variation (fluctuating/static) and their interaction on the above response variables (i.e. coralline algal growth rate, number of erect thalli, skeletal mineralogy, and diatom Chl *a*, BSi, and Redfield ratios) were separately tested using a two-way Analysis of Variance (ANOVA, *P* < 0.05) after homogeneity (Levene’s test) and normality (Shapiro-Wilk test) were satisfied. Significantly different groups were classified after Duncan’s Multiple Range Test (DMRT, *P* = 0.05). Statistical analyses were done using SPSS 18.0 (SPSS, Chicago, IL, USA).

## Results

### Coralline algae

**Fig 2:**
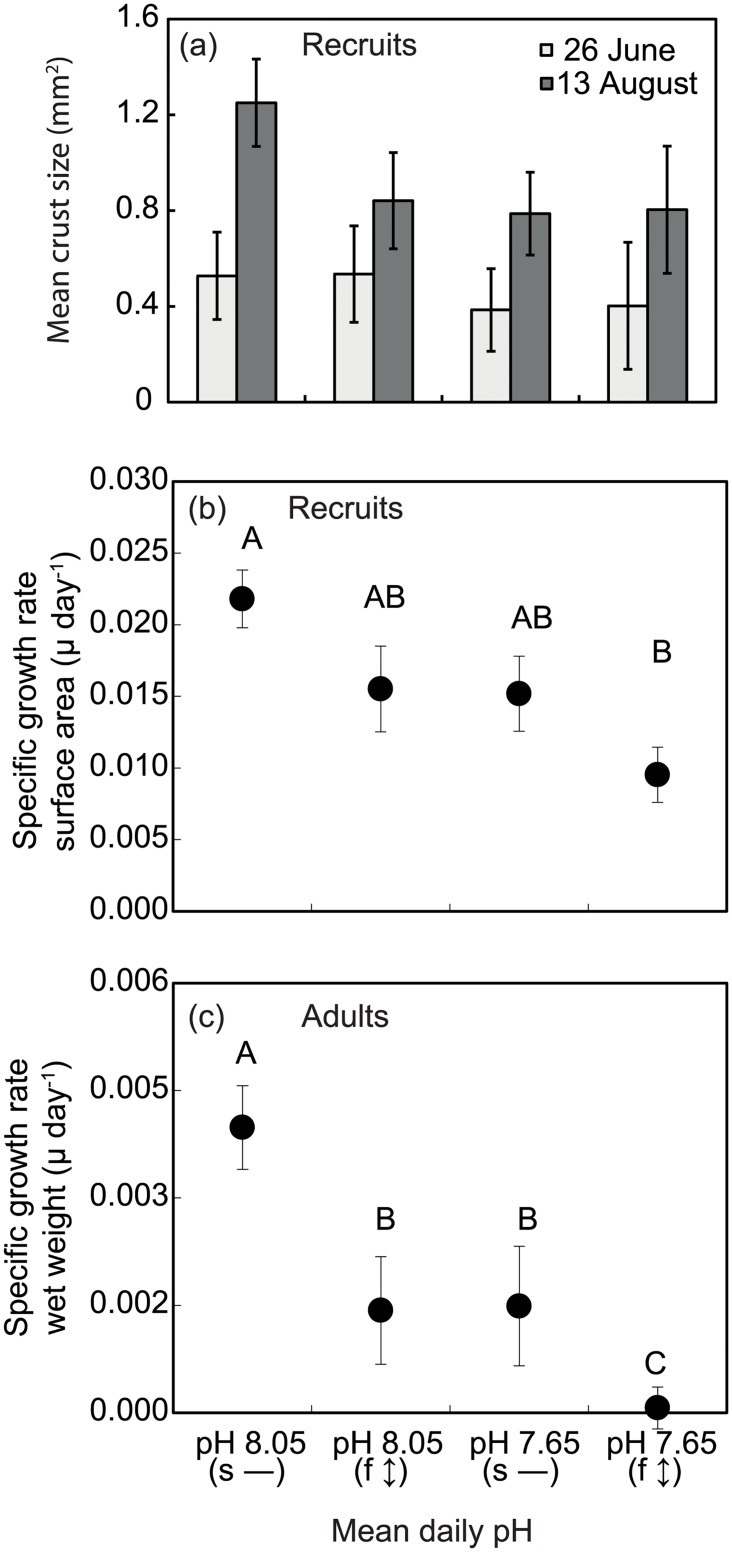
Spore development and growth rates of *Arthrocardia corymbosa*. (a) Crust size after nine (26^th^ June) and sixteen weeks (13^th^ August) in culture, and (b) Corresponding growth rates of the coralline algal recruits. (c) Growth rates (n = 6, ± SE) of the reproductive adults from Cornwall et al. [14] are re-plotted here to facilitate a direct comparison of the patterns in growth responses of adults vs recruits to static vs fluctuating pH. Experimental pH treatments (x-axis) were static (s—) and fluctuating (f ↕) pH conditions. Diurnally oscillating pH with mean daily pH 8.05 (f ↕) received pH-modified seawater of pH 8.45 during day and pH 7.65 at night, while mean daily pH 7.65 (f ↕) received pH-modified seawater of pH 8.05 during day and pH 7.25 at night. Static treatments received the same pH during day and night. In (b) and (c), points sharing the same letters are not significantly different (DMRT, *P>0*.*05*). c) Note that adult relative growth rate is on a dry weight basis whereas relative growth rates of new recruits (b) are on a surface area basis.

**Fig 3:**
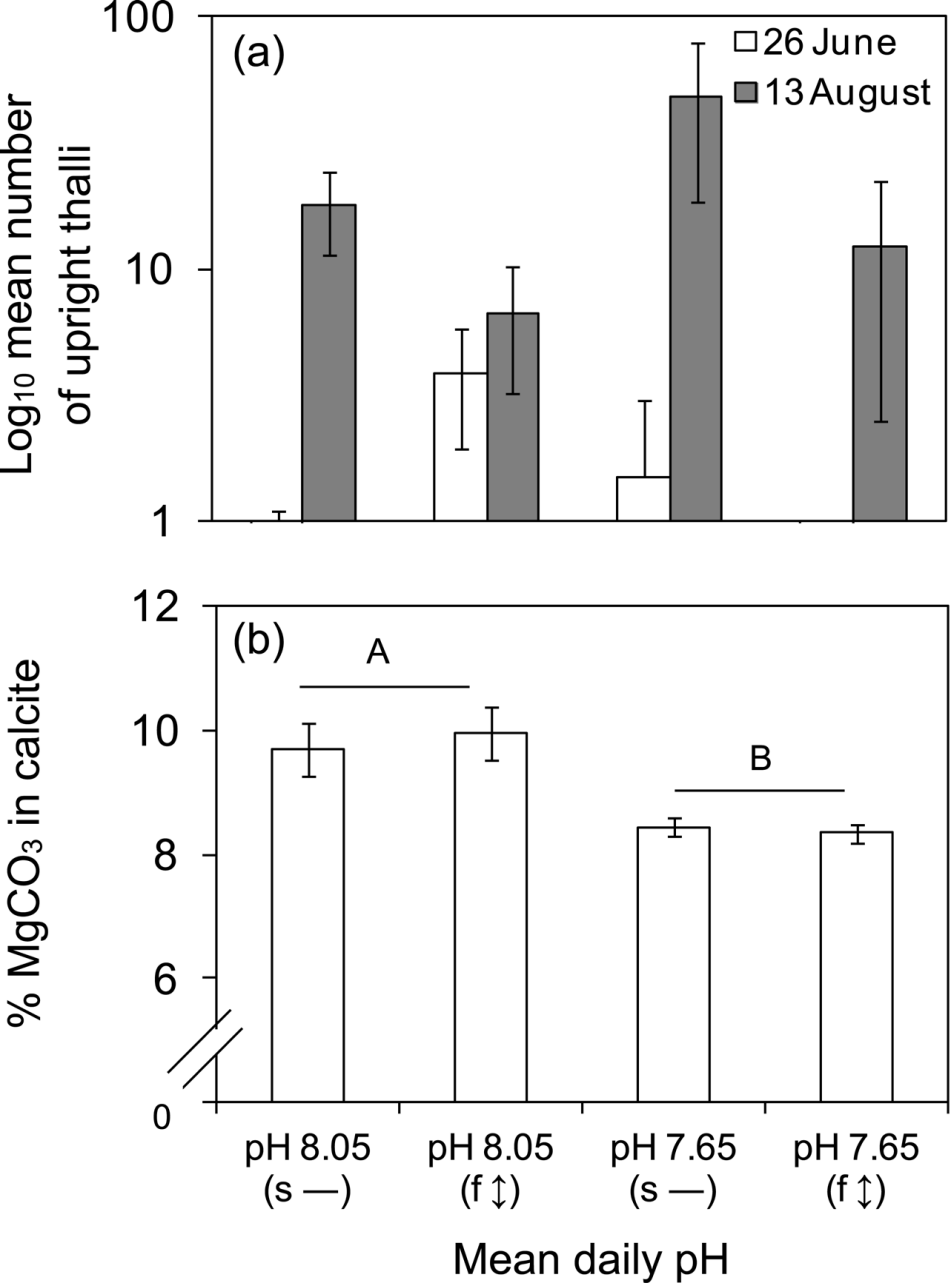
Number of fronds and skeletal mineralogy of *Arthrocardia corymbosa*. (a) Mean number of upright thalli (n = 6, ± SE) during two sampling periods, and (b) Corresponding skeletal mineralogy of the coralline algal recruits at the end of the experiment. The pH treatments were the same as in Fig 2. a) The main effects of pH and diurnal variation, as well as their interaction were not significantly different (ANOVA, *P* > 0.05). b) Percentage dry weight of MgCO_3_ in calcite was reduced by 14.5% under both low mean daily pH treatments. There was a statistically significant difference between pH (ANOVA, *P* = 0.016), but not between pH fluctuation (ANOVA, *P* = 0.804), nor their interaction (ANOVA, *P* = 0.255). Horizontal bar groupings and different letters refer to significant differences between mean values (n = 6, ± SE).

Under all experimental treatments, the spores of *Arthrocardia* germinated and grew into disc-shaped crusts before upright fronds were observed to develop (Fig 1c). After nine weeks, crust sizes were not significantly different between pH (*P* = 0.231), diurnal variation (*P* = 0.912) and their interaction (*P* = 0.964; Fig 2a; 26 June). At week 16, larger crusts were observed under static pH 8.05 (Fig 2a; 13 August), but the main effects of pH and diurnal variation, as well as their interaction, were not significantly different (*P* > 0.05). Within this latter 7-week period, the specific growth rate differed between pH (ANOVA, *P* = 0.020; DMRT, *P* = 0.05; pH 8.05 > pH 7.65) and diurnal variation (ANOVA, *P* = 0.027; DMRT, *P* = 0.05; static > fluctuation) but their interaction was not significantly different. Growth rate was highest under static pH 8.05 and lowest under fluctuating pH 7.65 (Fig 2b). Crust growth rate at μ = 0.009 was 37–56% lower under the fluctuating pH 7.65 relative to the other three treatments (μ = 0.015–0.022). At the end of the experiment, a greater number of upright thalli were observed under the static pH 7.65 ($\overset{-}{x}\;=\;48\;\pm 29\;SE)\;$(Fig 3a); 63–86% fewer upright thalli were observed in all other treatments (Fig 3a). Due to high variability between replicates, this difference was statistically non-significant between pH (ANOVA, *P* = 0.275), diurnal variation (ANOVA, *P* = 0.159) and their interaction (ANOVA, *P* = 0.450). Some bleaching of coralline recruits was noted across all treatments (S1 Fig) and there was no difference between the proportions of bleached individuals in any treatment. The percentage skeletal weight of MgCO_3_ in calcite was 14.5% lower in the pH 7.65 treatments compared to the pH 8.05 treatments (Fig 3b). The difference was statistically significant between pH (ANOVA, *P* = 0.016), but not between diurnal variation (ANOVA, *P* = 0.804), and their interaction (ANOVA, *P* = 0.255).

### Observations of fleshy seaweed recruitment

Turfing brown and green algae were observed in all treatments (S1 Table) and other fleshy brown macroalgae were found in all treatments except static pH 8.05 (S1 Table); identified were juvenile *Durvillea* sp., *Dictyota* sp. and *Desmarestia lingulata* (S1 Table, S2 Fig). Two species of fleshy red macroalgae were found in the fluctuating pH 8.05 treatment (S1 Table) and a crustose coralline species, *Synarthrophyton patena*, was also identified in this treatment (S1 Table, S2 Fig), which released tetraspores upon examination (S2c Fig). It should be noted that the specific species that arose in each culture container will depend on the spores available as epibionts on *Arthrocardia* thalli at the start of the experiment; we cannot therefore infer effects of OA or pH fluctuations.

### Benthic diatoms

**Fig 4:**
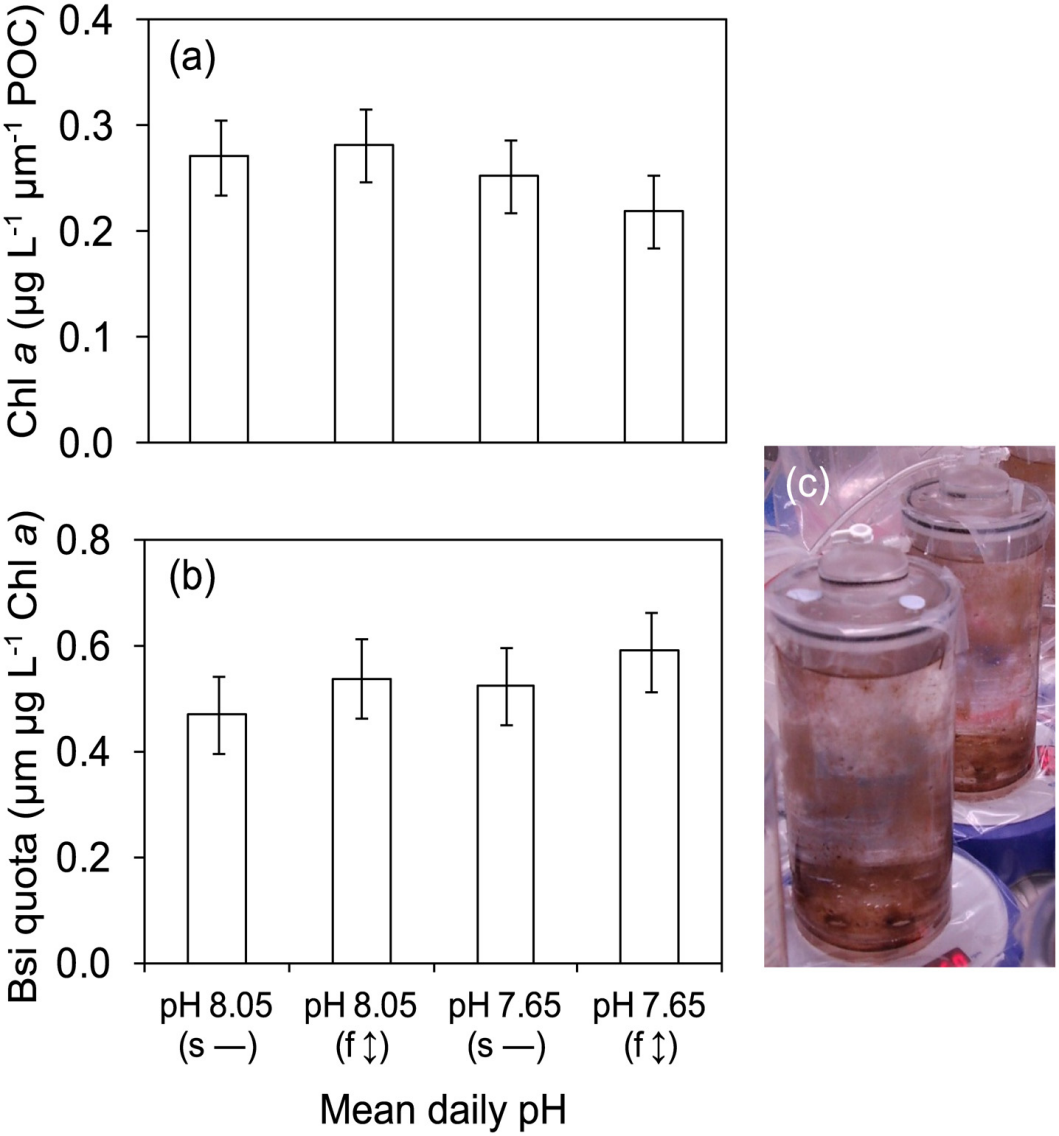
Responses of benthic diatom assemblage. (a) Chlorophyll *a*, and (b) Biogenic silica (both n = 6, ± SE) of (c) Benthic diatom assemblage in culture tanks. The pH treatments were the same as in Fig 2. a) and b) The main effects of pH and diurnal variation, as well as their interaction were not significantly different (ANOVA, *P* > 0.05).

At least six species of benthic diatoms, consisting of naviculoid (*Navicula sp*., *Fallacia sp*.), monoraphid (*Cocconeis sp*., *Achnanthes sp*.), and nitzschioid (*Nitzschia sp*., *Cylindrotheca sp*.), were observed across all treatments (S3 Fig). There was no effect of experimental treatment (i.e. pH, diurnal variation and their interaction; ANOVA, *P* > 0.05) on diatom community biomass (Chl *a*) or frustule silica content (BSi quota) (Fig 4a and 4b). Likewise, diatom stoichiometry (C:N, C:P, N:P, C:N:P) was not significantly different between treatments (S2 Table).

### Community metabolism and seawater carbonate chemistry

For each experimental treatment, the pH increased compared to the initial pH of the treatment after 4.4 h incubation at 18 μmol photons m^-2^ s^-1^ (Table 1). In static pH 7.65, pH increased by 0.15 (30% decreased in H^+^) after 4.4 h compared to static pH 8.05 where pH increased by 0.04 (9% decrease in H^+^). The 4.4 hourly seawater exchange in the culture chambers effectively exposed the community to persistent ambient (pH = 8.05–8.09) and ‘OA’ (pH = 7.65–7.80) seawater.

**Table 1:** Summary of seawater carbonate chemistry. Carbonate parameters (n = 6, ±SE) were calculated from total alkalinity (A_T_, n = 6) and pH (n = 6) measurements of seawater corresponding to each treatment. (+) and (–) refers to % increase and decrease, respectively, in carbonate chemistry parameters after 4.4h incubation under 18 μmol photons m^-2^ s^-1^.

Treatment	Cycle	Target pH	EXPM pH	H^+^ × 10^−3^ μmol kg^-1^	*p*CO_2_ μatm	A_T_ μmol kg^-1^	DIC μmol kg^-1^	H_2_CO_3_ μmol kg^-1^	${HCO}_{3}^{-}$ μmol kg^-1^	${CO}_{3}^{2-}$ μmol kg^-1^	ΩA	ΩC
Static pH 8.05	Night	8.05	8.05 (0.001)	9.00 (0.031)	430 (2)	2484 (11)	2285 (0.866)	19.32 (0.093)	2117 (1.313)	149 (0.536)	2.26 (0.008)	3.56 (0.013)
	Day	8.05	8.05 (0.004)	8.90 (0.084)	425 (6)	2484 (11)	2283 (2.381)	19.09 (0.250)	2114 (3.618)	151 (1.476)	2.29 (0.022)	3.59 (0.035)
	After 4.4h light incubation		8.09 (0.037)	8.08 (0.659)	356 (44)	2278 (55)	2068 (60.739)	15.98 (1.982)	1902 (64.372)	151 (13.295)	2.29 (0.202)	3.59 (0.317)
	% change		(+) 0.52	(-) 9.23	(-) 16.26	(-) 8.26	(-) 9.42	(-) 16.30	(-) 10.04	(+) 0.09	(+) 0.09	(+) 0.09
Fluctuating, mean daily pH = 8.05	Night	7.65	7.65 (0.005)	22.48 (0.233)	1180 (16)	2484 (11)	2439 (1.817)	52.11 (0.687)	2323 (1.921)	66 (0.783)	0.99 (0.012)	1.56 (0.019)
	Day	8.45	8.41 (0.003)	3.92 (0.028)	158 (2)	2484 (11)	2073 (2.664)	7.11 (0.079)	1779 (4.382)	288 (1.792)	4.37 (0.027)	6.85 (0.043)
	After 4.4h light incubation		8.34 (0.024)	4.61 (0.269)	183 (15)	2339 (12)	1990 (8.977)	8.25 (0.687)	1741 (20.598)	241 (12.969)	3.65 (0.197)	5.74 (0.309)
	% change		(-) 0.83	(+) 17.52	(+) 16.24	(-) 5.84	(-) 4.02	(+) 16.09	(-) 2.11	(-) 13.30	(-) 13.30	(-) 13.30
Static pH 7.65	Night	7.65	7.65 (0.002)	22.39 (0.094)	1174 (6)	2484 (11)	2438 (0.731)	51.88 (0.276)	2322 (0.774)	66 (0.315)	1.00 (0.005)	1.57 (0.008)
	Day	7.65	7.65 (0.001)	22.34 (0.039)	1172 (3)	2484 (11)	2438 (0.307)	51.78 (0.116)	2322 (0.325)	66 (0.132)	1.00 (0.002)	1.57 (0.003)
	After 4.4h light incubation		7.80 (0.029)	15.71 (1.110)	741 (68)	2271 (16)	2177 (18.110)	32.96 (2.978)	2061 (19.338)	84 (6.118)	1.28 (0.093)	2.00 (0.146)
	% change		(+) 2.00	(-) 29.71	(-) 36.78	(-) 8.56	(-) 10.71	(-) 36.35	(-) 11.24	(+) 27.47	(+) 27.47	(+) 27.47
Fluctuating, mean daily pH = 7.65	Night	7.25	7.23 (0.002)	59.03 (0.325)	3227 (26)	2484 (11)	2584 (1.383)	139.13 (1.090)	2420 (0.482)	26 (0.196)	0.39 (0.003)	0.62 (0.005)
	Day	8.05	8.05 (0.004)	8.88 (0.078)	424 (6)	2484 (11)	2282 (2.554)	19.04 (0.268)	2113 (3.881)	151 (1.584)	2.29 (0.024)	3.60 (0.038)
	After 4.4h light incubation		8.02 (0.036)	9.62 (0.821)	435 (62)	2279 (21)	2102 (37.684)	19.48 (2.748)	1954 (46.144)	130 (12.361)	1.98 (0.187)	3.10 (0.294)
	% change		(-) 0.43	(+) 8.27	(+) 2.49	(-) 8.25	(-) 7.89	(+) 2.26	(-) 7.56	(-) 13.72	(-) 13.72	(-) 13.72

The calculated aqueous CO_2_ was 171% higher under static pH 7.65 compared to static pH 8.05. After 4.4 h, the change in pH and A_T_ suggests 2.2× higher CO_2_ uptake under static pH 7.65 (37%) compared to static pH 8.05 (16%). However, the higher CO_2_ availability and assimilation under static pH 7.65 did not affect ${HCO}_{3}^{-}$ uptake (11%) which remains comparable to ${HCO}_{3}^{-}$ uptake (10%) under static pH 8.05 (Table 1).

Under fluctuating pH 8.05 and pH 7.65, communities previously exposed to night-time low pH 7.65 and pH 7.25 were subsequently exposed to seawater with a higher daytime experimental pH 8.41 (target pH = 8.45) and pH 8.05 (target pH = 8.05), respectively. During the first cycle under the light phase, the seawater pH decreased by 0.83% and 0.43% after 4 h under fluctuating pH 8.05 and fluctuating pH 7.65, respectively (Table 1).

## Discussion

The first hypothesis, that regardless of static or fluctuating pH the growth rates of the coralline recruits would be lower in pH 7.65 treatments compared to pH 8.05 treatments, was supported, and the second hypothesis that growth rates would be lower in fluctuating treatments than static was also supported. The growth responses of the newly recruited juvenile *Arthrocardia* to static and fluctuating pH followed the same pattern as those reported for the mature *Arthrocardia*, which were the ‘parental stock’ for the juveniles in this experiment [14]. The patterns are illustrated in Fig 2b and 2c, where the specific growth rates of adults (wet wt. basis) are plotted below those of the juveniles (surface area basis); while absolute differences in growth rate cannot be compared, the similarity of the pattern of growth responses to both pH and pH fluctuation is striking.

For the juveniles, however, the responses to mean pH and pH fluctuation treatments appear ‘less severe’ than those reported for the adults, as there was no significant difference between static pH 8.01, fluctuating pH 8.01 and static pH 7.65. Furthermore, in the fluctuating pH 7.65 treatment of Cornwall et al. [14], the adult growth rate was close to zero whereas for the juveniles a positive growth rate was recorded. This finding suggests that the juveniles were less severely affected by the experimental treatments than the adults.

Cold temperate coralline algae such as *Arthrocardia* that grow at the rock surface within kelp beds are naturally exposed to a wide range of pH, including pH 7.65, which is as low as the predicted average surface seawater pH for 2100 [14,23]. Hurd et al. [45] postulated that calcifying organisms naturally exposed to a wide range of pH may be better able to tolerate OA than those growing in a temporally constant pH. This was not the case for adult *Arthrocardia*, which had growth rates close to zero under the fluctuating pH 7.65 treatment (Fig 2c), in which a very low night-time seawater pH of 7.25 most likely contributed to partial dissolution of skeletal calcium carbonate [14]. The juvenile recruits of *Arthrocardia*, however, showed positive growth under this extreme environmental treatment (fluctuating pH 7.65, Fig 2b). This was surprising because early life-history phases are thought to be more susceptible to environmental stress factors, e.g. UVR [31,32] compared to their respective adult life stages.

There are two explanations as to why the juveniles maintained a positive growth rate in the most ‘severe’ experimental treatment (fluctuating pH 7.65) while the adults had a growth rate close to zero (Fig 2c and [14]). First, in this experiment the juveniles had periodic overgrowth of diatoms, which was removed every 2 weeks. These diatom films create a physical barrier between the calcifying coralline algal surface and the seawater. The pH at the surface of the crustose growth form of the juvenile coralline algae is therefore likely to be very different to that of the adults, as it will be modified by the diatom‘s metabolism [15]. The average pH at the surface of the crust-forming juveniles is therefore likely to be higher on average during a daily cycle, due to diatom photosynthesis, and this may moderate the very low night-time pH of 7.25.

A second, and not mutually exclusive, explanation is that of a positive carry-over effect on the next generation after exposing reproductive adults to an environmental stress i.e. OA. Such a positive effect has also been reported on the progeny of adult oysters grown in elevated *p*CO_2_ [46]. Likewise, meiospores of kelps releases from adults exposed to low-UV are more susceptible to UV-stress experiments compared to progeny of adults exposed to high UV [47]. Therefore, it is possible that the exposure of fertile *Arthrocardia* sporophytes to high/fluctuating *p*CO_2_ induced a preconditioning response to their spores to tolerate lower pH; these two ideas require experimental testing.

Cornwall et al. [14] was the first to experimentally simulate the pH fluctuations observed in nearshore kelp forest in the laboratory, and this study is the first to examine the responses of juvenile coralline algae to pH fluctuations. Flynn et al. [48] suggest that in a future ocean, algae will experience pH fluctuations, within the diffusion boundary layer (DBL) at their surface, that are larger than those previously experienced, because the buffering capacity of seaweed is reduced with reduced pH. Both Cornwall et al. [14] and this experiment on juveniles reveal that fluctuations strongly affect the response of corallines to OA compared to static treatments. Manipulating pH fluctuations is technically difficult, and in these first such experiments ([14] and this study), the pH changed suddenly from the daytime to night-time pH, and vice versa. In future experiments, more gradual changes would be ideal. It is, however, clearly important to consider pH fluctuations if we are to determine the influence of OA on coastal systems, many of which are dominated by strong diurnal pH signals [23].

Our third hypothesis, that experimental treatment would not affect the mineralogy of juvenile *Arthrocardia*, as reported by Cornwall et al. [14], was not supported. While diatoms can possibly increase pH at the surface of the crust forming juveniles, this would likely still not be sufficient to increase pH from 7.65 to 8.05 during daytime. Therefore, the decrease in Mg-calcite under pH 7.65 treatments compared to pH 8.05 suggests that the calcified structures of juvenile *Arthrocardia* recruits may be more porous and susceptible to dissolution compared to more compact adult skeletal structures. For example, low pH/high H^+^ weakens calcified structures in adult *Lithothamnion glaciale* [49]. Likewise, weaker calcite skeleton was observed in adult coralline algae exposed to low pH [50]. Species-specific changes in calcification rates, solubility, and density of calcite under ocean acidification have also been reported in other crustose coralline algae [51]. This study is the first to be able to compare the mineralogical responses to pH and pH fluctuations of juvenile coralline algal mineralogy with those of the field-collected adults, and further detailed studies are warranted.

Various fleshy algae recruited into our experiment, most likely germinated from cryptic propagules associated with and disentangled from the *Arthrocardia* thalli. We cannot make comparisons between treatments, as the results are descriptive, but it is of interest that a wide range of green, red and brown seaweeds recruited into each treatment, including the ‘severe’ pH 7.65 fluctuating treatment. The ability of these non-calcifying seaweeds to grow under these conditions suggest that they are tolerant to low pH, as also found for *Macrocystis pyrifera* and *Ulva rigida* [52–54], and fluctuating pH.

Our fourth (*a posteriori*) hypothesis, that diatom biomass will not be affected under all pH treatments, was supported. Diatoms are ubiquitous marine flora and their establishment inside our culture chambers most likely originated from epibiontic cells on the articulate thalli of adult *Arthrocardia* assemblage during the Cornwall et al. experiment [14]. The presence of an efficient carbon concentrating mechanism (CCM) in diatoms did not alter their performance under any pH treatment [55,56]. Moreover, diatoms also have membranes that are highly permeable to CO_2_ allowing a high flux of diffusive CO_2_ from the medium to the cell followed by active transport of carbon from the cytoplasm to the chloroplast [57]. As they have a high CO_2_ affinity, it is unsurprising that no negative effects of experimental treatments were recorded, even under fluctuating pH 7.65; this finding is consistent with James et al. [15]. Diatoms were observed to cover the inside of the culture chambers, both the Plexiglas side panels and the substrate (Fig 4c). In contrast, fleshy and coralline macroalgal recruits settled and grew only on the Plexiglas substrate, making the total cover of diatoms greater than that of fleshy and coralline macroalgae. The higher Δ CO_2_ under static pH 7.65 may be attributed to diatom CO_2_-uptake. Metabolism-mediated changes in seawater carbonate chemistry [45,58,59] may reduce the negative effects of OA in the future, increasing pH to a more suitable range for pH-sensitive species and promote diverse species community development.

Although the responses of diatoms to OA are reported to be species-specific, most studies either show similar [60–63] or enhanced [55,60,64,65] specific growth rates under elevated CO_2_. However, despite diatom’s ability to regulate internal acid-base balance, extremely low pH = 6.4 can negatively impact internal pH homeostasis and growth rate [66]. Likewise, medium alkalization due to high metabolic activity can have a negative effect. For example, an inverted U-shape relationship between pH and growth rate was observed in *Thalassiosira weissflogii*: growth rate was highest under an external pH (pHe) of 7.8 and lowest under extreme acidic (pHe 6.4) and basic (pHe 8.5) conditions [66]. On the other hand, acidification also modified the species’ intracellular silicic acid and biogenic silica (BSi) contents per cell. Unlike the inverted U-shape relationship between pH and growth rate, silica condensation and incorporation into the frustules was favoured under acidic and basic conditions giving a U-shape relationship between pH and silicic acid, and pH and BSi [66].

## Conclusions

Our study suggests that for juvenile *Arthrocardia*: (1) the growth rates are less sensitive to static pH 8.05 and pH 7.65 pH treatments compared to the same mean pHs with diurnal fluctuations, (2) exposure to fluctuating pH, especially to the extremely low night-time pH associated with the fluctuating pH 7.65 treatment, negatively affected their growth rate compared to static pH treatments, (3) the % weight of Mg Calcite in juveniles was significantly reduced at pH 7.65 compared to 8.05, and (4) diatom biomass was not affected by either pH or pH fluctuations.

Finally, some pH-sensitive species may survive lower pH conditions when they grow in the presence of diatoms and fleshy macroalgae, whose high metabolic activity may raise the pH of the local microhabitat. Moreover, the potential for acclimation and adaptation of early life history stages and first (F1) and second (F2) generation of offspring in response to acidification warrants further investigation.

## Supporting Information

S1 Fig *Arthrocardia corymbosa* recruits after 22 weeks’ cultivation. Recruits (a-c; g-i) under static and (d-f; j-l) under fluctuating pH conditions. Static treatments received pH 8.05 (a-c) and pH 7.65 (g-i) during day and night. Diurnally oscillating pH with mean daily pH 8.05 (d-f) received pH-modified seawater of pH 8.45 during day and pH 7.65 at night, while mean daily pH 7.65 (j-l) received pH-modified seawater of pH 8.05 during day and pH 7.25 at night. Scale bars = 2mm.
(TIF)

S2 Fig Fleshy and other coralline macroalgal recruits. Genera, species and functional groups associated with juvenile *Arthrocardia corymbosa* recruits as summarized in S1 Table: (a) foliose red, (b) filamentous red, (c) discoid and warty *Synarthrophyton patena*, (d) green and brown turfs among young *A*. *corymbosa* upright frond with crustose base, (e) *Durvillaea* sp., (f) *Dictyota* sp., (g) *Desmarestia lingulata*, indicated by an arrow, and (h) brown thread-like filaments. Scale bars = 2mm, except (c) and (h), scale bar = 1mm.
(TIFF)

S3 Fig Images of diatoms taken with a scanning electron microscope (SEM). Multi-species benthic diatom assemblage consisting of two or more of the below were observed under all pH treatments. (a) *Fallacia* sp., (b) *Achnanthes* sp. 1, (c) *Cocconeis* sp. 1, (d) *Cocconeis* sp. 2, (e) *Achnanthes* sp. 2, (f) *Navicula* sp., (g) naviculoid species, (h) *Cylindrotheca* sp., (i) nitzschoid species, (j) dividing *Nitzschia* sp. 1, (k) dividing *Navicula* sp., and (l) *Nitzschia* sp. 2.
(EPS)

S1 Table Observations of macroalgae that recruited into the experimental culture tanks during the experiment. (+) indicates the presence of a particular functional group and/or genus/species that grew within an individual replicate culture chamber/tank (numbered 1–24) that was associated with one of the four experimental treatments.
(DOCX)

S2 Table Stoichiometry of benthic diatom biomass under the four experimental treatments (see Methods). Values in parentheses are standard error (± SE; n = 6).
(DOCX)
