# Supplementary figures and images for: Effect of Ocean Acidification and pH Fluctuations on the Growth and Development of Coralline Algal Recruits, and an Associated Benthic Algal Assemblage
Source: PLoS One. 2015 Oct 15;10(10):e0140394. doi: 10.1371/journal.pone.0140394 (PMC4607452; doi:10.1371/journal.pone.0140394)

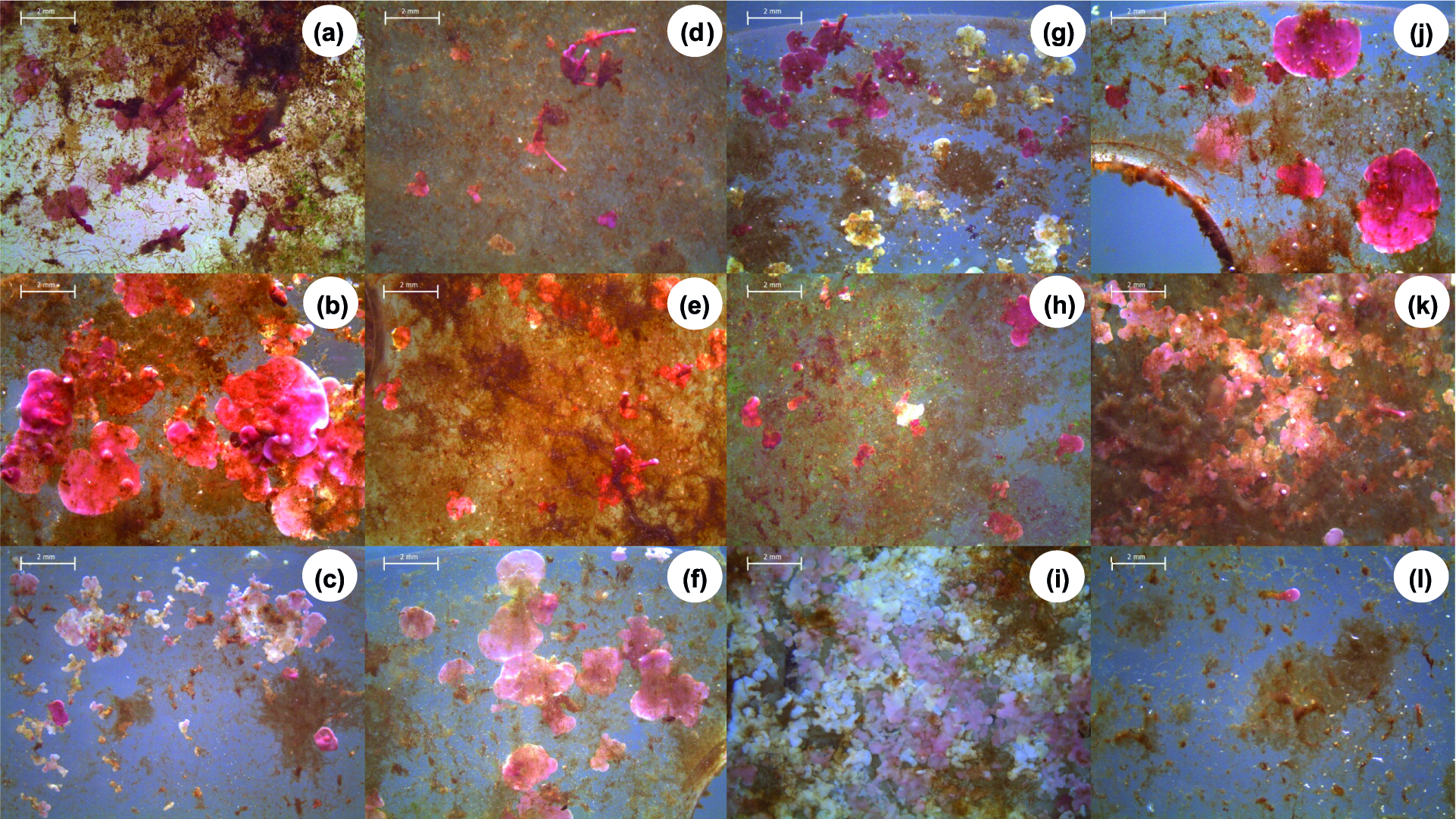

Supplement: S1 Fig — Recruits (a-c; g-i) under static and (d-f; j-l) under fluctuating pH conditions. Static treatments received pH 8.05 (a-c) and pH 7.65 (g-i) during day and night. Diurnally oscillating pH with mean daily pH 8.05 (d-f) received pH-modified seawater of pH 8.45 during day and pH 7.65 at night, while mean daily pH 7.65 (j-l) received pH-modified seawater of pH 8.05 during day and pH 7.25 at night. Scale bars = 2mm. (TIF) [file pone.0140394.s001.tif]

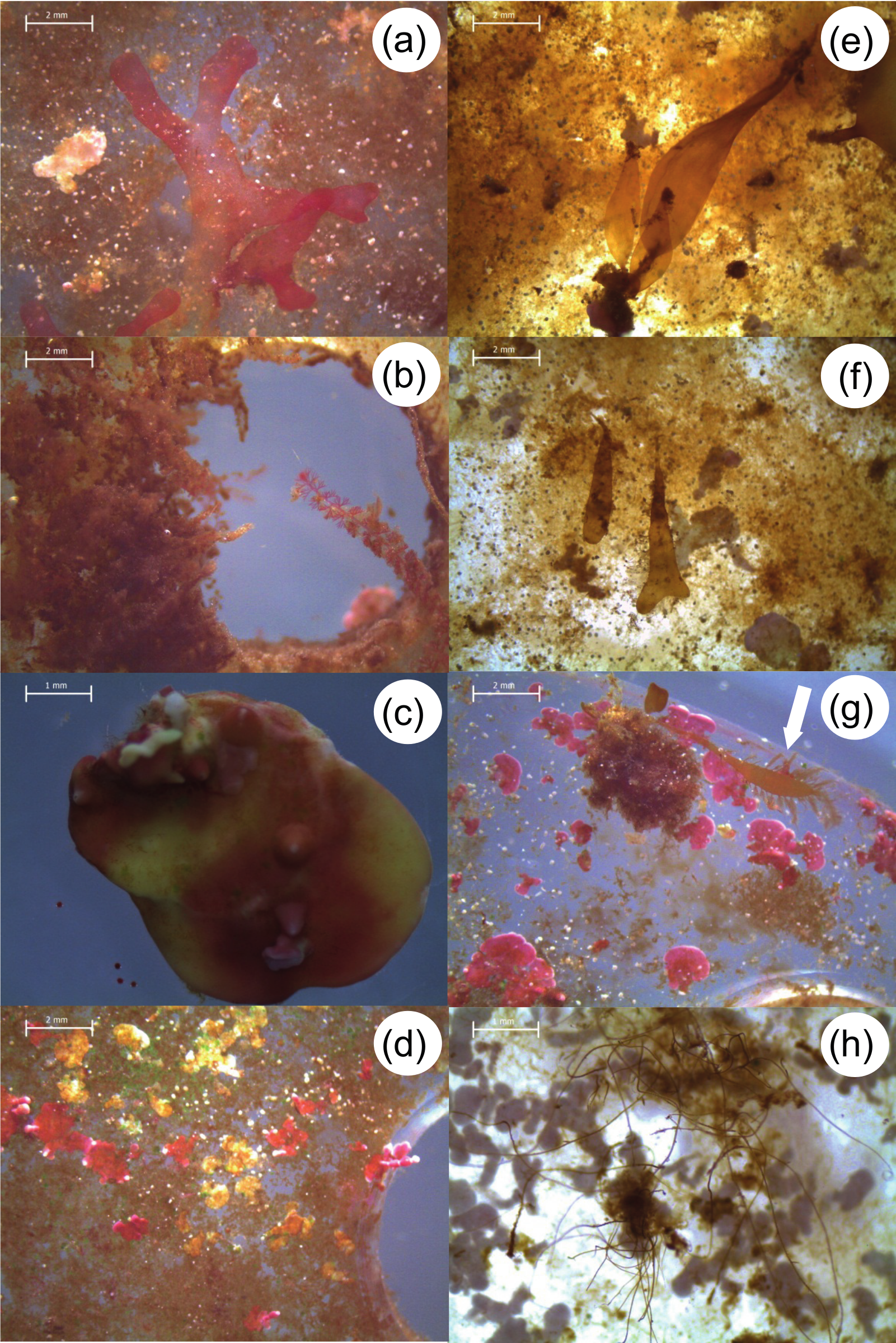

Supplement: S2 Fig — Genera, species and functional groups associated with juvenile Arthrocardia corymbosa recruits as summarized in S1 Table: (a) foliose red, (b) filamentous red, (c) discoid and warty Synarthrophyton patena, (d) green and brown turfs among young A. corymbosa upright frond with crustose base, (e) Durvillaea sp., (f) Dictyota sp., (g) Desmarestia lingulata, indicated by an arrow, and (h) brown thread-like filaments. Scale bars = 2mm, except (c) and (h), scale bar = 1mm. (TIFF) [file pone.0140394.s002.tiff]
