# Supplementary material for: Effect of Ocean Acidification and pH Fluctuations on the Growth and Development of Coralline Algal Recruits, and an Associated Benthic Algal Assemblage
Source: PLoS One. 2015 Oct 15;10(10):e0140394. doi: 10.1371/journal.pone.0140394 (PMC4607452; doi:10.1371/journal.pone.0140394)
Supplement: S1 Table — (+) indicates the presence of a particular functional group and/or genus/species that grew within an individual replicate culture chamber/tank (numbered 1–24) that was associated with one of the four experimental treatments. (DOCX) [file pone.0140394.s004.docx]

**Supplementary Tables**

**S1 Table.** Observations of macroalgae that recruited into the experimental culture tanks during the experiment. (+) indicates the presence of a particular functional group and/or genus/species that grew within an individual replicate culture chamber/tank (numbered 1-24) that was associated with one of the four experimental treatments.

| Treatment | Culture tank number | Other recruits | | | | | | | | |
| --- | --- | --- | --- | --- | --- | --- | --- | --- | --- | --- |
|  |  | Turf- green | Turf- brown | *Durvillaea* sp. | *Desmarestia lingulata* | Brown- foliose  (*Dictyota* sp.) | Brown- filaments | Red- foliose | Red- filamentous | Other coralline  (*Synarthrophyton patena*) |
| Static pH 8.05 | 2 | + | + |  |  |  |  |  |  |  |
|  | 5 |  | + |  |  |  |  |  |  |  |
|  | 9 |  | + |  |  |  |  |  |  |  |
|  | 15 | + | + |  |  |  |  |  |  |  |
|  | 18 | + | + |  |  |  |  |  |  |  |
|  | 21 |  | + |  |  |  |  |  |  |  |
| Fluctuating  mean pH= 8.05  daytime pH=8.45 nighttime pH= 7.65 | 6 |  | + | + | + | + |  | + |  |  |
|  | 10 |  | + |  |  |  |  |  | + |  |
|  | 12 |  | + | + |  |  |  |  |  | + |
|  | 14 | + | + | + |  |  |  |  |  |  |
|  | 23 |  | + |  |  |  |  |  |  |  |
|  | 24 |  | + |  | + |  |  |  |  |  |
| Static pH 7.65 | 1 |  | + |  |  |  |  |  |  |  |
|  | 4 |  | + |  |  |  |  |  |  |  |
|  | 7 |  | + |  |  |  | + |  |  |  |
|  | 13 | + | + | + | + |  |  |  |  |  |
|  | 17 | + | + |  |  |  |  |  |  |  |
|  | 20 | + | + |  |  |  |  |  |  |  |
| Fluctuating  mean pH= 7.65  daytime pH=8.05 nighttime pH= 7.25 | 3 |  | + |  |  |  |  |  |  |  |
|  | 8 |  | + |  |  |  |  |  |  |  |
|  | 11 |  | + |  |  |  |  |  |  |  |
|  | 16 |  | + |  |  |  |  |  |  |  |
|  | 19 | + | + | + |  |  |  |  |  |  |
|  | 22 |  | + |  | + |  |  |  |  |  |
