# Supplementary material for: Effect of Ocean Acidification and pH Fluctuations on the Growth and Development of Coralline Algal Recruits, and an Associated Benthic Algal Assemblage
Source: PLoS One. 2015 Oct 15;10(10):e0140394. doi: 10.1371/journal.pone.0140394 (PMC4607452; doi:10.1371/journal.pone.0140394)
Supplement: S2 Table — Values in parentheses are standard error (± SE; n = 6). (DOCX) [file pone.0140394.s005.docx]

**Supplementary Tables**

**S2 Table.** Stoichiometry of benthic diatom biomass under the four experimental treatments (see Methods). Values in parentheses are standard error (± SE; n=6).

| Treatments | C:N | C:P | N:P | C:N:P |
| --- | --- | --- | --- | --- |
| Static, mean pH 8.05 | 8.67:1 (± 0.6) | 198:1 (± 4) | 23:1 (± 4) | 198:23:1 |
| Fluctuating, mean pH= 8.05 | 8.68:1 (± 1.1) | 204:1 (± 15) | 25:1 (± 8) | 204:25:1 |
| Static, mean pH 7.65 | 8.52:1 (± 0.4) | 210:1 (± 23) | 25:1 (± 6) | 210:25:1 |
| Fluctuating, mean pH= 7.65 | 8.87:1 (± 0.5) | 209:1 (± 21) | 23:1 (± 3) | 209:23:1 |
